# The role of tonsillectomy in the Periodic Fever, Aphthous stomatitis, Pharyngitis and cervical Adenitis syndrome; a literature review

**DOI:** 10.1186/s12901-017-0049-5

**Published:** 2018-02-22

**Authors:** Jostein Førsvoll, Knut Øymar

**Affiliations:** 1 Department of Paediatrics, Stavanger University Hospital, PO BOX 8100, 4068 Stavanger, Norway; 2 Department of Clinical Science, University of Bergen, Bergen, Norway

**Keywords:** PFAPA, Tonsils, Tonsillectomy, Adenotonsillectomy

## Abstract

**Background:**

Tonsillectomy (TE) or adenotonsillectomy (ATE) may have a beneficial effect on the clinical course in children with the Periodic Fever, Aphthous stomatitis, Pharyngitis and cervical Adenitis (PFAPA) syndrome. However, an immunological reason for this effect remains unknown. This literature review summarizes the current knowledge of the effect of TE or ATE in the PFAPA syndrome.

**Methods:**

A search of PubMed, Medline, EMBASE and Cochrane was conducted for papers written in English dated from 1 January 1987 to 31 December 2016. The search included all studies reporting outcomes after TE or ATE from children aged 0 to 18 years with PFAPA.

**Results:**

Two randomized controlled trials reported significantly faster resolution of febrile episodes after TE or ATE in children with PFAPA compared to controls (non-surgery groups). We identified 28 case series including 555 children with PFAPA. The diagnosis was set prospectively before surgery in 440 children and retrospectively after surgery in 115 of the children. TE or ATE had a curative effect in 509 of the 555 children with PFAPA (92%), but few studies were of high quality.

**Conclusion:**

TE or ATE may have a curative effect on children with PFAPA, but the evidence is of moderate quality. Further high-quality randomized controlled studies are still needed.

**Electronic supplementary material:**

The online version of this article (10.1186/s12901-017-0049-5) contains supplementary material, which is available to authorized users.

## Background

The Periodic Fever, Aphthous stomatitis, Pharyngitis and cervical Adenitis (PFAPA) syndrome is the most common paediatric periodic fever syndrome [1, 2], with a cumulative incidence of 2.2 per 10.000 children up to the age of 5 years in a Nordic population [2]. The hallmarks of the disease are short (3–5 days), regularly occurring episodes of high fever accompanied by at least one of the following major symptoms: pharyngitis, cervical adenitis and aphthous stomatitis [3–5]. The febrile episodes are accompanied by a marked inflammatory response with C-reactive protein >100 mg/L with complete normalization between the episodes [6]. Episodes of PFAPA often start during the first few years of life and often spontaneously resolve during late childhood [2, 7].

PFAPA has not been defined genetically, and the aetiology is unknown. A dysregulated interleukin-1 response may play a part in the aetiology of the disease [8, 9], and PFAPA is currently regarded as an autoinflammatory disease [10]. There is no established international consensus regarding the definition of PFAPA [11]. The clinical entity was first described by Marshall et al. in 1987 [12], and in 1989 they presented the acronym PFAPA and suggested a set of diagnostic criteria for the syndrome [13]. In 1999, Thomas et al. presented a modified set of diagnostic criteria and since then these criteria have been widely used in international studies [4]. The criteria by Marshall and Thomas are principally equivalent, but in addition to excluding cyclic neutropenia, the definition by Marshall et al. also systematically excludes rare hereditary periodic fever syndromes.

As early as in 1989, Abramson et al. reported that in four children with PFAPA, fever episodes ceased after tonsillectomy (TE) [14]. Since then, the outcome after TE or adenotonsillectomy (ATE) has been reported in several case series of children with PFAPA, indicating a beneficial effect of surgery on the clinical course. Based on two randomized controlled studies, a recent Cochrane review concluded that the evidence for the effect of TE in children with PFAPA is of moderate quality [15], but the case series have not been systematically described before.

There is currently no other curative treatment for children with PFAPA, and for those with bothering symptoms highly influencing daily life for the child and family, TE or ATE has become an option to consider [2, 3, 15].

This literature review summarizes the results of all studies reporting the outcome of TE or ATE in children with the PFAPA syndrome.

## Methods

A systematic search of the PubMed, Medline, EMBASE and Cochrane databases was performed up to January 31th 2017 using the keywords “Marshall Syndrome”, “PFAPA” and “Periodic Fever, Aphthous stomatitis, Pharyngitis and cervical Adenitis”. Papers in English language published between 1 January 1987 and 31 December 2016 were checked for relevance, and the references in the relevant papers were also reviewed to identify any articles not found in the systematic search. Papers were included if they reported the outcome after TE or ATE in children aged 0 to 18 years diagnosed with PFAPA. The diagnostic criteria for the diagnosis in each study were noted; according to Marshall [12, 13], Thomas [4] or adapted criteria not clearly based on either of the two.

The outcome after TE or ATE was defined as “curative” if a cessation of febrile episodes occurred; as “partly effective” if the children experienced less frequent, shorter or less severe symptoms during subsequent febrile attacks; and as “not effective” if the disease pattern remained unchanged. The study design was reviewed, and registered if the PFAPA diagnosis was set prospectively before surgery or retrospectively after surgery had been performed. When concurrence of authorship and time, the studies were thoroughly assessed for overlap of patients included.

If a case series included ≥20 patients, a follow-up of ≥24 months and the diagnosis was set prior to surgery, the study was considered as high quality. If two of these criteria were present the study was considered of moderate quality, and with ≤ one criteria of low quality. High quality study should have included patients according to definitions by Marshall or Thomas. The review was performed according to the Additional file 1: PRISMA guidelines [16].

## Results

**Fig. 1:**
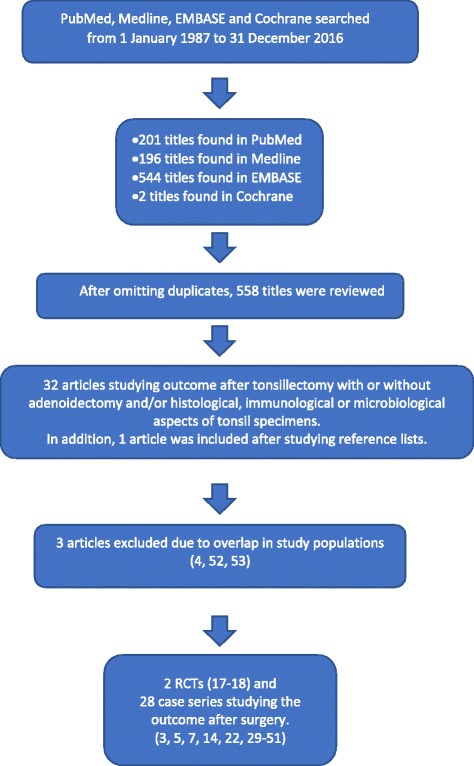
Overview of the systematic literature search [52, 53]

After omitting duplicates, the search retrieved 558 manuscripts (Fig. 1). After a review of the titles, abstracts, and reference lists of the relevant manuscripts, two randomized controlled trials (RCTs) and 28 case series reporting the outcome after TE or ATE in children with PFAPA were identified. Two Cochrane studies were identified, but no other systematic reviews were found.

### The outcome of PFAPA after tonsillectomy with or without adenoidectomy

The first RCT performed by Renko et al. included 26 children with PFAPA (mean age: 4.1 years) [17]. They found that tonsillectomy was curative in all 14 children randomized to the TE group, whereas six of the 12 children in the control group experienced spontaneous resolution of PFAPA episodes within 6 months after inclusion in the study (*p* < 0.001).

In the RCT by Garavello et al., 39 children with PFAPA were randomized to either ATE (*n* = 19) or expectant management (*n* = 20) groups [18]. Twelve of the children in the ATE surgery group had a prompt resolution of symptoms (63%), whereas only one child in the control group experienced spontaneous resolution during the 18 months of follow-up (5%) (*p* < 0.001). The mean number of episodes recorded during the follow-up period was 0.7 (1.2; SD) in the surgery group and 8.1 (3.9) in the control group (p < 0.001). The episodes were further described as less severe in the surgery group.

As summarized in Table 1, the outcome after TE or ATE has been reported in 28 case series, including a total of 555 children with PFAPA. The diagnosis was set prospectively before surgery in 450 children and retrospectively after surgery in 115 of the children. Three studies were categorized as high quality, six as medium quality and 19 as low quality. Surgery was curative in 509 children (92%), partly effective in 14 children and not effective in 32 children. Surgery was curative in 160 of 176 children (91%) in studies with low quality and in 149 of 161 children (93%) in studies with moderate quality. In the high quality studies, surgery was curative in 200 of 218 children (92%). In 16 of the studies, the mean time of observation after surgery was sufficiently given, with a median time of 19 months (11, 26.5) (interquartile range).

**Table 1:** Case series reporting the outcome after tonsillectomy and adenotonsillectomy in children with the PFAPA syndrome

	Author/year (reference)	Number of children	Curative	Partly effective	Not effective	Observation post surgery (months, mean)	Adeno-tonsillectomy^a^	Diagnosis set prior to surgery	Criteria for PFAPA diagnosis^b^	Quality
1	Abramson et al. 1989 [14]	4	4	–	–	15	3 (75%)	No	Marshall	Low
2	Padeh et al. 1999 [3]	3	3	–	–	Not clear	0	Yes	Marshall	Low
3	Dahn et al. 2000 [30]	5	5	–	–	3	5 (100%)	No	Adapted	Low
4	Galanakis et al. 2002 [31]	15	15	–	–	Not clear	0	No	Thomas	Low
5	Berlucchi et al. 2003 [32]	5	5	–	–	10	2 (40%)	Yes	Adapted	Low
6	Parikh et al. 2003 [33]	2	0	–	2	Not clear	0	No	Marshall	Low
7	Tasher et al. 2006 [5]	6	6	–	–	19	0	Yes	Thomas	Low
8	Wong et al. 2008 [34]	9	8	1	–	24	0	Yes	Marshall	Medium
9	Pignataro et al. 2009 [35]	9	5	4	–	26	0	Yes	Marshall	Medium
10	Fedrer et al. 2010 [36]	11	11	–	–	18	0	Yes	Thomas	Low
11	Peridis et al. 2010 [37]	9	8	–	1	12	2 (22%)	Yes	Thomas	Low
12	Wurster et al. 2011 [7]	12	6	3	3	Not clear	10 (83%)	Yes	Thomas	Low
13	Licameli et al. 2012 [38] ^c^	102	99	–	3	43	102 (100%)	Yes	Marshall	High
14	Førsvoll et al. 2013 [29]	17	17	–	–	Not clear	7 (41%)	Yes	Thomas	Low
15	Krol et al. 2013 [39]	18	18	–	–	Not clear	0	Yes	Thomas	Low
16	Ter Haar et al. 2013 [40]	8	4	3	1	Not clear	Not clear	Yes	Marshall	Low
17	Valenzuela et al. 2013 [41]	9	9	–	–	10	0	Yes	Thomas	Low
18	Kubota et al. 2014 [42]	5	4	–	1	Not clear	0	Yes	Thomas	Low
19	Vigo et al. 2014 [43]	41	27	–	14	69	0	Yes	Marshall	High
20	Dytrych et al. 2015 [44]	10	10	–	–	19	0	Yes	Thomas	Low
21	Førsvoll et al. 2015 [45]	4	4	–	–	27	1 (25%)	Yes	Thomas	Medium
22	Lantto et al. 2015 [46]^d^	31	31	–	–	6	11 (35%)	No	Adapted	Low
23	Perko et al. 2015 [47]	28	26	2	–	Not clear	0	Yes	Thomas	Medium
24	Batu et al. 2016 [48]^c^	53	50	–	3	Not clear	0	Yes	Thomas	Medium
25	Dusser et al. 2016 [49]	4	3	–	1	Not clear	Not clear	Yes	Thomas	Low
26	Erdogan et al. 2016 [50]	75	74	1	–	24	Not clear	Yes	Thomas	High
27	Lantto et al. 2016 [22]^d^	58	56	–	2	107	0	No	Thomas	Medium
28	Rigante et al. 2016 [51]	2	1	–	1	Not clear	0	Yes	Thomas	Low
	Total	555	509	14	32	–	143 (26%)	–		

*PFAPA* Periodic Fever, Aphthous stomatitis, Pharyngitis and cervical Adenitis

^a^:Adenotonsillectomy performed instead of tonsillectomy alone

^b^:PFAPA diagnosis was set according to criteria by Marshall [13], Thomas [4] or adapted criteria not clearly based on either of the two

^c^and ^d^:Possible overlap between study populations

Two Cochrane reviews by Burton et al. in 2010 and 2014 studied the clinical effectiveness of TE or ATE compared to nonsurgical treatment in the management of PFAPA [15, 19], both reviews based on the two randomized studies only. They concluded that TE appears to be a useful treatment option in the management of children with PFAPA syndrome, with moderate-quality evidence.

## Discussion

The two RCTs performed indicate a beneficial effect of TE or ATE in children with PFAPA. The study by Renko et al. had a short time of follow-up, and has also been criticized for vague diagnostic criteria not according to Marshall or Thomas [20, 21]; the large percentage of children described as having PFAPA but with fever as their only symptom may be the reason for the speculation concerning the specificity of the PFAPA diagnosis in this study. A clear and uniform definition should be applied to compare results from studies and provide generalizability to patient fulfilling these criteria. However, in a recent publication from this study group, they showed that TE may also be effective as a treatment for children with recurring febrile episodes who do not meet the classical diagnostic criteria for PFAPA [22].

Garavello et al. did a thorough diagnostic workup of all children included in their RCT, they applied strict diagnostic criteria for PFAPA, and there was a longer time of follow-up. They performed ATE on all children who underwent surgery and showed favourable outcomes for these children compared with the control group. Therefore, this study may serve as the best evidence for TE or ATE in children with PFAPA.

Case series have important limitations and provide the lowest level of evidence. A beneficial outcome after TE or ATE in 523 of 555 children is impressive. However, only three studies were considered by us as high quality. In one of these studies, TE was curative in only 66% of patients, but in 92% of patients in all the high quality studies together. Moreover, subgroups of patients may have been selected for the operations, and publication bias may be another important limitation for case series. Several of the case series lack information regarding follow-up or have a brief period of follow-up. Therefore, a firm conclusion on the effect of TE or ATE cannot be drawn based on these studies.

It is not clear if ATE is more effective than TE alone. In the highest-quality study included in this review, the RCT by Garavello et al., ATE was performed in all children. Further studies are needed to confirm these findings and bring clarity to this issue.

Tonsillectomy for PFAPA has also been described in adult patients with PFAPA, but the results are not included in this review. The data are limited, but the procedure seems to be less effective for adults than for children [23–26].

Taken together, a high rate of success of TE or ATE has been shown in two RCTs, of which one with high quality, and in case series with high and moderate quality. In our opinion the current literature therefore supports TE or ATE as a treatment option for children with PFAPA, but with a moderate level of evidence. However, the disease is benign with a high likelihood of spontaneous resolution during childhood [2, 5, 7]. In one study of 59 children with PFAPA, the mean duration of symptoms before resolution was 6.3 years [7], whereas in one study of 46 children, the median age of resolution differed by only 10 months between those who were operated or not [2]. The indication for surgery must therefore be evaluated separately in each child based on the burden of the disease, the impact of recurrent febrile episodes on the family, and signs of impending resolution, such as less-severe and shorter febrile episodes and longer afebrile intervals [2, 5]. The decision must be taken by physicians and parents together based on the degree of symptoms and the best possible knowledge of different outcomes.

Several studies have indicated that a dysregulated immune system may play a part in the aetiology of PFAPA [8, 9, 27–29]. In 2011, Stojanov et al. proposed a model for PFAPA where a microbial trigger initiates a cascade leading to the febrile attacks. They suggested that an immunologically immature host or a host with an inherited or acquired immune abnormality plays a permissive role [8]. However, the observation that removal of the tonsils, which serve as a minor part of the secondary immune system, may be curative for a disease that is possibly caused by a dysregulated immune system remains puzzling.

## Conclusion

In conclusion, two RCTs and several case series indicate that TE or ATE has a beneficial effect on the course of PFAPA, but the evidence is of moderate quality. Surgery must be weighed against the chance of spontaneous recovery. More RCTs on the effect of TE with strict diagnostic criteria for PFAPA are needed.

## Additional file

Additional file 1: PRISMA guidelines checklist (DOC 63 kb)

## Abbreviations

ATE: Adenotonsillectomy
PFAPA: Periodic fever, aphthous stomatitis, pharyngitis and cervical adenitis
RCT: Randomized controlled trial
TE: Tonsillectomy
